# The Complex Roles of Plant Hormones During Clubroot Disease Development in the Brassicaceae

**DOI:** 10.1007/s00344-025-11790-9

**Published:** 2025-08-01

**Authors:** Charitha P. A. Jayasinghege, Emilee R. M. Storfie, Jocelyn A. Ozga, Stephen E. Strelkov

**Affiliations:** 1 Agassiz Research and Development Centre, Agriculture and Agri-Food Canada, Agassiz, BC Canada; 2 Plant BioSystems, Department of Agricultural, Food and Nutritional Science, University of Alberta, Edmonton, AB Canada

**Keywords:** *Plasmodiophora brassicae*, Auxin, Cytokinins, Salicylic acid, Jasmonic acid, Abscisic acid, Ethylene

## Abstract

Clubroot, caused by the obligate parasite *Plasmodiophora brassicae*, is a serious soilborne disease that threatens many commercially valuable crops in the Brassicaceae family, including the oilseed crop canola (*Brassica napus*) and various vegetables. Evidence from studies analyzing hormonal profiles, transcriptomes, proteomes, mutants defective in hormone functions, and treatments of infected plants with growth regulators suggest that nearly all plant hormones are involved in or affected by the disease. However, the specific roles of individual hormones in clubroot development or resistance remain unclear. This knowledge gap is compounded by the complex regulation of hormone functions and inconsistencies across studies, likely due to variations caused by host–pathogen combinations and other factors such as environmental influences. Additionally, biotic and abiotic stress responses caused by the disease and, in some instances, pathogen proteins manipulating host hormonal metabolism add additional layers of complexity. Despite these challenges, emerging trends suggest regulatory roles for plant hormones in both disease development and host defense. In this review, we explore these patterns, aiming to elucidate the contributions of different hormones to clubroot development and associated stress responses.

## Introduction

Clubroot, caused by the obligate parasite *Plasmodiophora brassicae*, is a soilborne disease that affects plants in the Brassicaceae (crucifer) family. Characterized by club-shaped root galls, the disease hinders water and nutrient absorption, resulting in stunted growth, reduced yield, and in severe cases, even death of the plant (Dixon 2009). The disease has been a longstanding concern for farmers, with historical records tracing back to 1736, when it was identified as a serious and contagious disease affecting turnip crops in England (Wellman 1930). Nearly a century and a half later, Russian scientist M. S. Woronin began studying the disease and identified its causal agent, *P. brassicae*, in 1878 (Dixon 2009). Today, clubroot is a globally spread disease that affects many economically valuable Brassicaceae crops (Dixon 2009, 2014). One of the most impacted is canola or oilseed rape (*Brassica napus*), a major oil crop representing a multibillion-dollar industry (Dolatabadian et al. 2022). Other important crops affected include *B. oleracea* varieties, such as cabbage, broccoli, cauliflower, kale, and kohlrabi; *B. rapa* varieties such as turnip and Chinese cabbage; other *B. napus* varieties including rutabaga and mustard; and numerous other *Brassica* species (Dixon 2009).

**Fig. 1:**
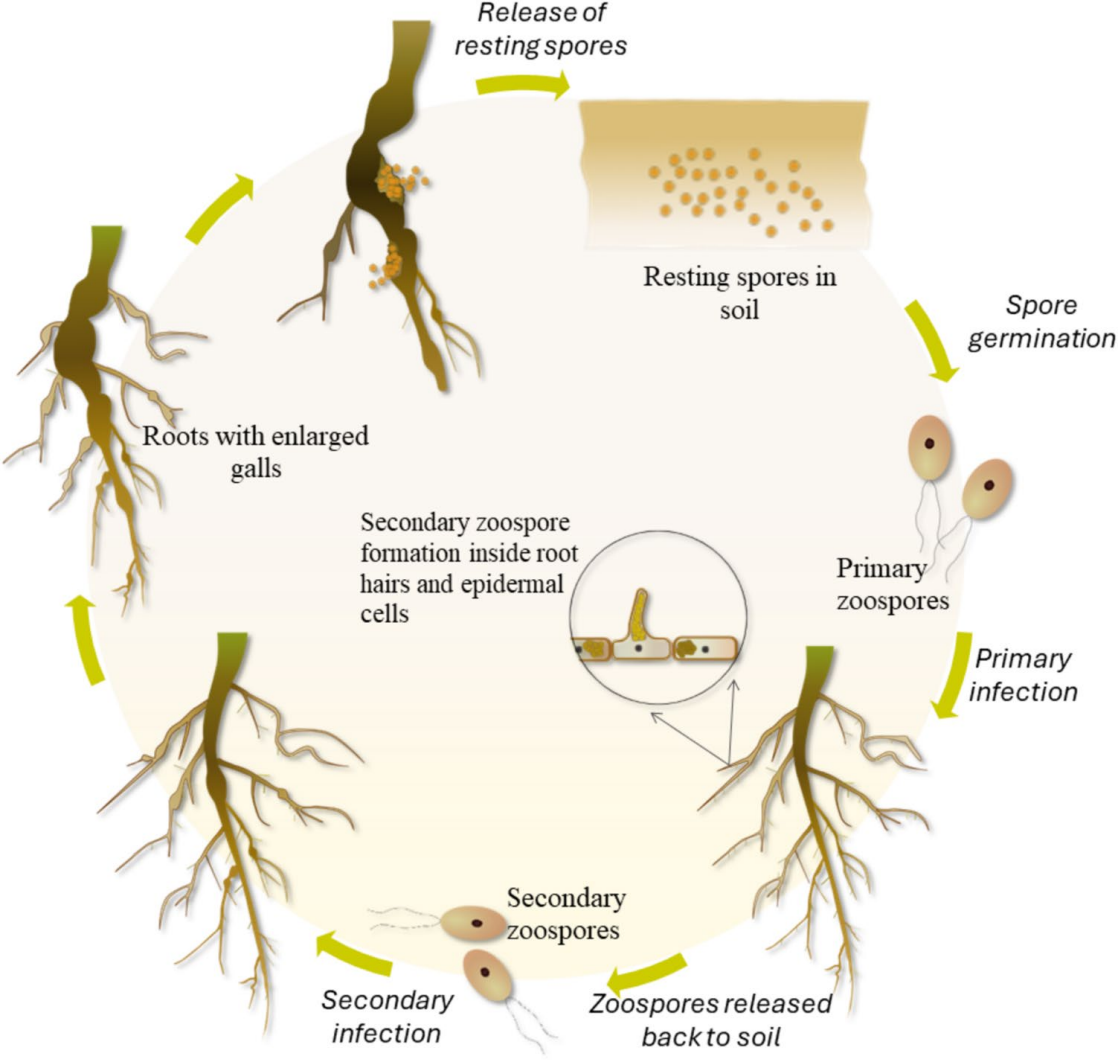
The clubroot disease cycle. Resting spores of *Plasmodiophora brassicae* germinate to release primary zoospores. These zoospores infect plant root hairs and epidermal cells, forming primary plasmodia that develop into zoosporangia, giving rise to secondary zoospores. The secondary zoospores are released back into the soil and reinfect the roots. During this stage, the pathogen infects the root cortex, inducing host cell division and enlargement, which results in the formation of root galls. Inside the infected roots, *P. brassicae* produces intracellular secondary plasmodia, which eventually cleave to produce a new generation of resting spores. These are released back into the soil as the root galls disintegrate

The pathogen *P. brassicae* is a soilborne obligate biotroph, historically categorized as a fungus but later reclassified as a protist within the Phytomyxea group of the Rhizaria supergroup (Bulman and Braselton 2014; Hwang et al. 2012). Its infection cycle begins when resting spores in the soil germinate, releasing primary zoospores that infect host root hairs and epidermal cells (Fig. 1). This initial infection, known as the primary infection, does not cause the formation of root galls and has been observed even in some non-hosts (Liu et al. 2020a). Inside root hairs, the pathogen forms primary plasmodia, which develop into zoosporangia where secondary zoospores are produced. These zoospores are released back into the soil and subsequently infect the root cortex. This secondary infection produces intracellular secondary plasmodia and causes excessive host cell division and enlargement, resulting in root galls (Liu et al. 2020b; Ludwig-Müller and Schuller 2008). Eventually, the secondary plasmodia produce a new generation of resting spores, with an estimated 10 billion spores per gram of root gall in *B. napus,* capable of surviving in soil for up to 15–20 years (Hwang et al. 2013; Wallenhammar 1996).

The biotrophic nature of the clubroot pathogen and the hardiness of its spores make chemical control largely ineffective (Auer and Ludwig-Müller 2023). Therefore, prevention and cultivation of resistant plants are the primary disease management strategies (Saharan et al. 2021). However, qualitative resistance, typically controlled by a single resistance gene, is often short-lived, as selection pressure leads to new pathotypes capable of overcoming this resistance (Dolatabadian et al. 2022; Drury et al. 2021; Strelkov et al. 2016, 2018). Consequently, quantitative resistance based on multiple minor-effect genes holds more potential for developing new cultivars (Dolatabadian et al. 2022). Understanding how the pathogen overcomes plant defense and promotes symptom development can also provide additional avenues for improving resistance or suppressing disease severity. A key component in this regard is an understanding of plant hormonal action during clubroot development, as the pathogen likely manipulates hormones to overcome host defenses and promote gall formation (Jayasinghege et al. 2023; Ludwig-Müller et al. 2015; Ludwig-Müller and Schuller 2008).

We will review the literature on plant hormones during clubroot disease development, focusing on auxins, gibberellins (GAs), cytokinins (CKs), abscisic acid (ABA), ethylene, jasmonates (JAs), salicylic acid (SA), and brassinosteroids (BRs). Since the disease triggers growth stimulation to generate root galls, pathogen defense activation, and abiotic stress mitigation to cope with reduced water uptake, the plant hormonal balance is likely disrupted in multiple ways. This complexity, together with overlapping roles and interactions among hormones, makes evaluating hormonal regulation challenging (Jayasinghege et al. 2023; Ludwig-Müller et al. 2009; Ludwig-Müller and Schuller 2008; Prerostova et al. 2018; Vañó et al. 2023). Nonetheless, extensive work has been conducted over the years to understand the role of plant hormones during clubroot development.

In addition to studies on plant hormone interactions, microarray and RNA sequencing (RNAseq) analyses provide indirect insights and are the primary sources for most of the gene expression data discussed here. While quantitative PCR (qPCR) is required for high sensitivity and accuracy in determining the expression patterns of specific genes (Horta et al. 2023), these expression analyses enable broader predictions of gene expression changes. Although a diverse array of treatment comparisons is available (Shaw et al. 2022; Wang et al. 2022a; Wei et al. 2021; Zhang et al. 2016), to reduce complexity, we primarily focus on those comparing inoculated and non-inoculated plants of the same cultivar at the same time-point when discussing gene expression. Discrepancies among studies, even within the same host species or cultivar, are also common. These differences may arise from spatiotemporal differences in hormone action, complex interactions among hormones, and genetic or environmental factors influencing pathogen establishment. While these challenges complicate drawing definitive conclusions, our aim is to summarize the current understanding of the roles of different hormones in clubroot disease development and associated plant defense and stress responses.

## Auxin

**Fig. 2:**
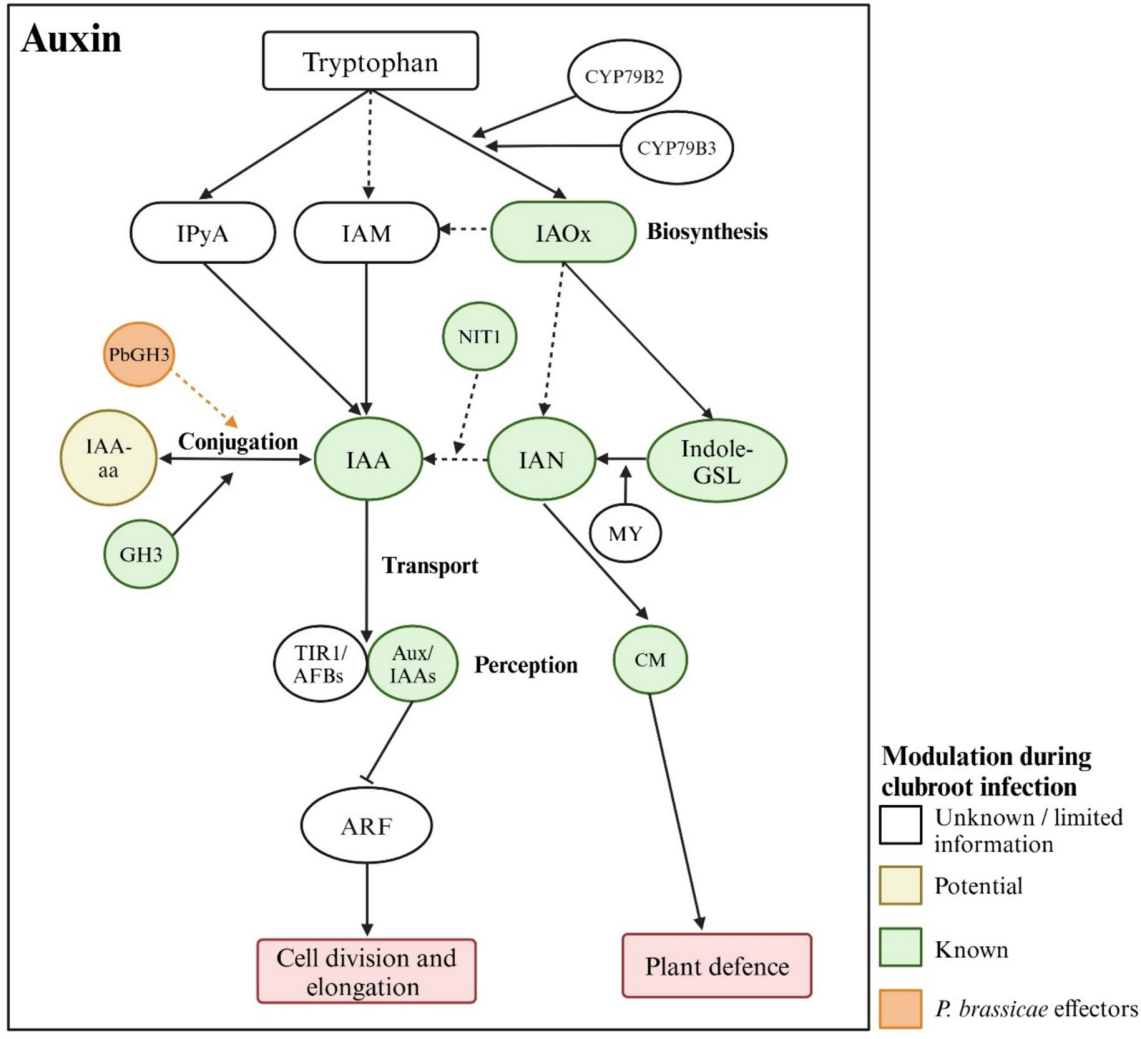
Modulation of auxin pathways during clubroot disease development. Indole-3-acetic acid (IAA) is primarily synthesized from tryptophan (Trp) via multiple possible pathways, including indole-3-pyruvic acid (IPyA), indole-3-acetaldoxime (IAOx), and indole-3-acetamide (IAM) pathways. Clubroot development may be promoted through the IAOx pathway by stimulation of *NIT* gene expression, which converts indole-3-acetonitrile (IAN) into IAA. IAOx also produces indole glucosinolates (indole-GSL), which are broken down by myrosinases (MY) to produce defense compounds like camalexin (CM). However, *cyp79b2 cyp79b3* double mutants, which lack detectable levels of IAOx, show no difference in clubroot susceptibility, suggesting a limited role for this pathway. IAA levels can be regulated by conjugation, including the formation of amino acid conjugates (IAA-aa) by GH3 amido synthetases. While *P. brassicae* harbors the *GH3* homolog *PbGH3*, it does not appear to influence host IAA conjugation or disease development significantly. TIR1/AFBs and Aux/IAAs function as auxin receptors. The binding of auxin triggers the degradation of Aux/IAA repressors, thereby derepressing ARF transcription factors, which regulate the expression of auxin-responsive genes. Pointed arrows indicate stimulation, while flat-tipped arrows represent inhibition. Solid and dashed lines denote well-defined and poorly defined processes, respectively

Auxin, a central regulator of many plant processes, including cell division and elongation (Perrot-Rechenmann 2010), has long been suspected to play a role in clubroot disease (Butcher et al. 1974). Indole-3-acetic acid (IAA), the ubiquitous bioactive form of auxin, is primarily synthesized from the precursor tryptophan (Trp) through multiple potential pathways, classified by their intermediates (Fig. 2). The most well-characterized and predominant is the indole-3-pyruvic acid (IPyA) pathway, where Trp is converted to IPyA by TRYPTOPHAN AMINOTRANSFERASE OF ARABIDOPSIS 1 (TAA1) and TAA1-RELATED aminotransferases (TARs), and then into IAA by the YUCCA (YUC) family of flavin monooxygenases (Casanova-Sáez et al. 2021; Zhao 2014). However, so far, changes in the IPyA pathway have not been linked to root gall formation. Similarly, the IAM (indole-3-acetamide) pathway has not shown clear links to gall formation. Most evidence associating modulation of the auxin pathways with clubroot disease development is through the IAOx (indole-3-acetaldoxime) pathway.

As in the case of clubroot disease, the IAOx auxin biosynthesis pathway appears to be limited to Brassicaceae plants (Casanova-Sáez et al. 2021). In this pathway, Trp is converted to IAOx by the cytochrome P450 (CYP) isozymes CYP79B2 and CYP79B3. IAOx may be further converted into indole-3-acetonitrile (IAN) or IAM to subsequently produce IAA, although the enzymes involved in these conversions are not fully identified (Casanova-Sáez et al. 2021). Additionally, IAOx can be transformed into indole glucosinolates and camalexin, which play roles in plant defense against pathogens (Casanova-Sáez et al. 2021). The breakdown of indole glucosinolates by myrosinases also generates the IAA precursor IAN. This dual role in auxin biosynthesis and plant defense suggests that the IAOx pathway may facilitate both symptom development and plant defense in the clubroot pathosystem (Ludwig-Müller 2009).

When *P. brassicae*-infected root segments were infiltrated with ^14^C-labeled Trp, synthesis and metabolism of indole-3-methylglucosinolate and IAN increased, suggesting that infected plants produce higher levels of these potential IAA precursors (Rausch et al. 1983). The conversion of IAN to IAA is believed to be regulated by the NIT1-subfamily of nitrilases, consisting of NIT1-NIT3 (Casanova-Sáez et al. 2021; Lehmann et al. 2017). In Arabidopsis, *NIT1* antisense lines exhibited reduced gall formation, while *NIT2* antisense lines exhibited delayed gall formation (Neuhaus et al. 2000). Additionally, *NIT1*- and *NIT2*-promoter::β-glucuronidase reporter constructs showed increased promoter activities toward the later stages of gall formation (Grsic-Rausch et al. 2000). However, while recombinant proteins (from *B. rapa* expressed in *Escherichia coli*) of NIT1 and NIT2 were able to convert IAN to IAA, IAN was a poor substrate (Ishikawa et al. 2007), and a lack of direct genetic evidence has left their role in IAA biosynthesis uncertain (Casanova-Sáez et al. 2021). Moreover, in Arabidopsis, *cyp79b2 cyp79b3* double mutants that produce no detectable levels of IAOx (Sugawara et al. 2009) showed no difference in clubroot susceptibility, indicating that IAOx pathway-derived IAA or the defense compounds are not essential for gall formation or clubroot defense (Siemens et al. 2008). Nevertheless, a comparison of susceptible and partially resistant Arabidopsis cultivars revealed a stronger increase in camalexin in the resistant interactions, and increased susceptibility in a mutant (*pad3*) defective in camalexin biosynthesis. This suggests that the IAOx pathway may still play a contributing role in clubroot defense (Lemarié et al. 2015b).

Multiple hormone profiling studies have investigated changes in IAA profiles in response to root gall formation. Studies monitoring steady-state levels of IAA during clubroot development show considerable variability in the literature, and in general, there is a lack of reports on IAA turnover. For instance, in *B. rapa*, one study found no change in free IAA levels at 6 and 13 days after inoculation (DAI) but a reduction at 21 DAI compared to non-inoculated controls (Devos et al. 2005). Another study observed reduced IAA levels at 3 and 9 DAI, with no significant change at 20 DAI in inoculated susceptible plants (Wei et al. 2021). Similarly, in *B. napus*, one study reported increased IAA levels in susceptible interactions at 3 and 7 DAI with no change at 10 DAI (Xu et al. 2016), while another found reduced IAA levels at 4 and 21 DAI, with no change at 14 DAI (Jayasinghege et al. 2023). There was also no change in IAA and IAA conjugate levels in *P. brassicae*-infected Arabidopsis roots with hypocotyls when analyzed at 4 DAI (Devos et al. 2006).

While increased IAA levels are more frequently reported approximately 3 weeks post-inoculation in Arabidopsis (Grsic-Rausch et al. 2000; Ludwig-Müller et al. 1999) and in *B. rapa* (Grsic et al. 1999; Ludwig-Müller et al. 1996; Ugajin et al. 2003), observations across time-points indicate fluctuating IAA levels rather than consistently higher levels during gall formation. These fluctuations may reflect the ability of the pathogen to maintain optimal plant IAA levels for symptom development. Alternatively, these hormonal profiles may not fully capture the actual changes in IAA levels associated with root gall formation, which could be localized to specific sections or cells of the roots undergoing gall formation. For example, the expression analysis of IPyA pathway genes indicates highly tissue-localized auxin biosynthesis in certain developmental processes (Schaller et al. 2015). Therefore, more tissue- and developmental-specific analysis using laser-capture microdissection or IAA-responsive reporter gene expression lines, along with continued quantification of IAA levels and turnover during gall development, would provide deeper insights into the metabolic flux through the IAA pathway and provide a more robust measure of IAA biosynthesis and metabolism during clubroot development.

In addition to biosynthesis, steady-state auxin levels within a tissue are influenced by metabolic processes that include oxidation and conjugation, as well as auxin transport (Zhang and Peer 2017). Gene expression studies in Arabidopsis and Chinese cabbage (*B. rapa* ssp. *pekinensis*) using qPCR, RNAseq, or microarray analyses show that multiple members of the Gretchen Hagen 3 (GH3) family, a central group of enzymes responsible for conjugating IAA, JA, and SA to amino acids, exhibit changes in expression, primarily showing up-regulation during disease development (Jahn et al. 2013; Ludwig-Müller 2014; Robin et al. 2020; Schuller et al. 2014). Notably, genome sequencing of *P. brassicae* revealed a *GH3* homolog in its genome (Rolfe et al. 2016; Schwelm et al. 2015) that, despite not closely resembling plant GH3s, can conjugate both IAA and JA in vitro (Schwelm et al. 2015; Smolko et al. 2024). However, overexpression of *PbGH3* in Arabidopsis neither significantly affected hormone levels nor altered clubroot susceptibility, suggesting that it plays no or a limited role in disease development (Smolko et al. 2024).

Differential expression of the auxin transport genes PIN-FORMED1 (PIN1), PIN-LIKES1 (PILS1), and AUXIN-RESISTANT1/LIKE AUX1 (AUX1/LAX1) has also been reported in *P. brassicae*-infected Chinese cabbage (Robin et al. 2020). Application of the auxin transport inhibitor N-1-naphthylphthalamic acid reduced disease severity and altered the localization of root galls in *B. napus*, while application of IAA increased disease severity (Xu et al. 2016). These observations and changes in auxin levels indicate that plants actively modulate IAA levels in infected roots, including through conjugation and transport.

Cells detect and respond to changes in auxin levels primarily through a signaling cascade initiated at the TRANSPORT INHIBITOR RESPONSE 1/AUXIN SIGNALING F-BOX (TIR1/AFB) and AUXIN/INDOLE-3-ACETIC ACID (Aux/IAA) family of coreceptors (Fig. 2). IAA acts as a “molecular glue” to bring together TIR1/AFBs and Aux/IAAs. This interaction leads to the ubiquitination and subsequent degradation of Aux/IAAs (Calderon-Villalobos et al. 2010). In the absence of auxin, Aux/IAAs repress the activity of the AUXIN RESPONSE FACTORs (ARFs) that regulate the expression of auxin-responsive genes. The degradation of Aux/IAAs derepresses ARFs, enabling their regulation of auxin-responsive genes (Yu et al. 2022).

The TIR1/AFB auxin receptors in Arabidopsis consist of six members: TIR1 and AFB1-AFB5. TIR1 appears to be the primary receptor in root growth, followed by AFB2, with other receptors playing contributing roles. AFB1, however, is an outlier that does not seem to participate in canonical auxin signaling but may contribute to a rapid auxin response (Parry et al. 2009; Prigge et al. 2020). When *tir1* and *afb1-1* single mutants and *afb1-1afb2-3* double mutants were tested for clubroot susceptibility, all showed increased susceptibility at lower inoculum densities (Jahn et al. 2013). Conversely, the IAA17 mutant *auxin-resistant 3-1* (*axr3-1*), which lacks root hairs and displays an impaired auxin response akin to increased auxin sensitivity (Leyser et al. 1996), showed reduced clubroot susceptibility (Alix et al. 2007). These observations are, however, not sufficient to conclude an inverse correlation between auxin sensitivity and clubroot susceptibility. For instance, given that the roles of AFB1 and TIR1 in canonical auxin signaling are drastically different, it is not immediately clear if the increased susceptibility of both *tir1* and *afb1* mutants results from reduced auxin sensitivity. The extent to which the absence of root hairs influences the reduced susceptibility in the *axr3-1* mutant is also unclear. Additional evaluations of other receptor mutants, including higher-order mutants, are needed to determine whether changes in auxin sensitivity correlate with clubroot severity.

Overall, research on auxin biosynthesis, homeostasis, signaling, and plant auxin responses suggests modulation of auxin during clubroot disease development. However, studies have yet to establish direct links between auxin biosynthesis and gall formation, and investigations into auxin action have been limited to a small number of mutants, offering insufficient evidence for its role in root gall formation. The complexity of multiple auxin biosynthesis pathways and the overlapping functions of genes involved in auxin homeostasis and action have complicated the establishment of solid links. Further studies, such as higher-order mutant evaluations, temporal and spatial auxin steady-state level and turnover profiling, and more targeted analysis of auxin activity in infected tissues, are needed to understand the role of auxin in clubroot disease. Ultimately, identifying the extent of the contribution of various pathways that regulate IAA levels and signaling during *P. brassicae* infection and clubroot disease progression could lead to new strategies for managing resistance, as it is not the pathogen itself but the associated gall formation that hinders root functions and weakens plant performance.

## Cytokinins

CKs are a class of phytohormones primarily known for stimulating cell division and modulating cell differentiation (Li et al. 2021). Common active CKs include trans-zeatin (tZ), isopentenyladenine (iP), cis-zeatin (cZ), and dihydrozeatin (DZ), with tZ and iP showing more potent biological activity and central roles in CK function in Arabidopsis and many other plant species (Osugi and Sakakibara 2015). An early study found that callus tissue from *P. brassicae*-infected cabbage plants grew in a CK-free growth medium, whereas calli from uninfected plants did not, suggesting increased CK biosynthesis or activity in response to infection (Reddy and Williams 1970). In Arabidopsis, iP level and the CK-responsive *ARABIDOPSIS RESPONSE REGULATOR 5* (*ARR5*) promoter activity increased early in disease development in infected roots and subsequently spread to the hypocotyl and upper root system, indicating elevated CK activity (Devos et al. 2006; Siemens et al. 2006). In canola, high levels of cZ were reported in the roots of a clubroot-susceptible cultivar during early disease development, while a partially resistant cultivar showed no change or a decline. Other CKs, including tZ, DZ, and iP, showed no clear trend, while cZ levels were either lower or unchanged at later time-points after inoculation (Prerostova et al. 2018). These data indicate that clubroot development correlates with higher CK levels, particularly preceding gall formation.

**Fig. 3:**
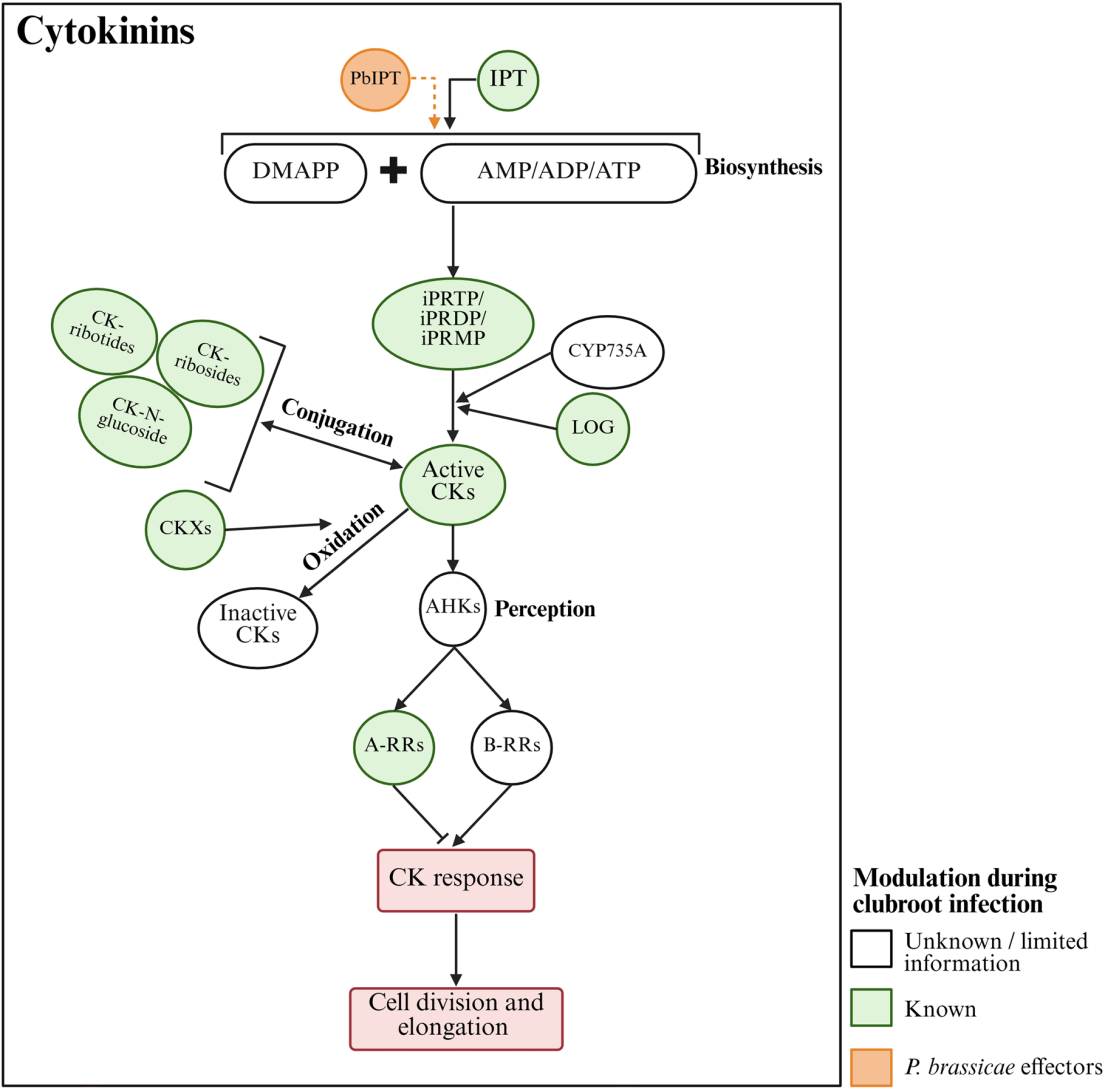
Cytokinins (CKs) and clubroot disease. CK biosynthesis is primarily catalyzed by IPTs, which form CK-nucleotides (iPRTP, iPRDP, and iPRMP) from dimethylallyl pyrophosphate (DMAPP) and adenosine phosphates (AMP, ADP, or ATP). The CK-nucleotides are converted into active CKs directly through a process catalyzed by the enzyme LOG or through an intermediate step catalyzed by CYP735A. Increased CK levels and *IPT* expression, especially during early clubroot disease development, suggest a role for CKs in pathogenesis. Although *P. brassicae* has two *IPT* homologs, there is no strong evidence to suggest their involvement in host CK biosynthesis. CK levels are regulated by oxidation catalyzed by CKXs and reversible conjugation. CK perception by AHKs triggers a phosphorylation cascade that activates Type-A and Type-B response regulators (RRs). Type-B RRs promote CK-responsive gene expression. In some infected plants, reduced expression of *CKX* and increased expression of *RRs* further support heightened CK activity. Pointed and flat-tipped arrows indicate stimulation and inhibition, respectively. Solid lines denote well-defined processes, whereas dashed lines indicate poorly defined processes

In the primary route of CK biosynthesis, ISOPENTENYL TRANSFERASE (IPT) uses 5-phosphate adenosine (AMP, ADP, and ATP) and dimethylallyl pyrophosphate (DMAPP) to produce cytokinin nucleotides (iPRTP, iPRDP, and iPRMP). These compounds are converted into CKs directly through a process catalyzed by the enzyme LONELY GUY (LOG; cytokinin riboside 5′-monophosphate phosphoribohydrolase that releases cytokinin nucleobase and ribose 5′-monophosphate), or via an intermediate step catalyzed by Cytochrome P450 monooxygenase 735 A (CYP735A) (Kurakawa et al. 2007; Osugi and Sakakibara 2015; Fig. 3). Active CK levels can be regulated through irreversible oxidation by CYTOKININ OXIDASEs (CKXs) producing biologically inactive metabolites, and through conjugation by cytokinin glycosyltransferases. Free CK forms can also be reversibly converted into biologically inactive CK-glucosides, as well as the inactive precursors CK-ribotides and CK-ribosides (Osugi and Sakakibara 2015; Wu et al. 2021).

A Northern blot analysis of *IPT1* genes in Chinese cabbage found increased transcript abundance of several genes in symptom-free regions of the root at 20 DAI, where galls would later develop, while their transcript abundance was lower than controls in regions with visible galls later in disease development (Ando et al. 2005). In Arabidopsis, where the disease cycle typically progresses faster than in crops like canola or Chinese cabbage, *IPT1* and *IPT7* were upregulated in the upper 1 cm section of the root system at 16 and/or 26 DAI. However, *IPT3* and *IPT5*, the most strongly expressed *IPT* genes in this tissue, and several *LOG* genes showed downregulations (Malinowski et al. 2016). The root-hypocotyl sections of these plants also contained lower levels of tZ and iP, as well as CK-ribosides, CK-nucleotides, and CK N-glucoside (Malinowski et al. 2016). Similar results were also reported in a subsequent study at 28 DAI (Bíbová et al. 2023). Another study reported a reduction in root tZ and its riboside levels as early as 12 DAI (Blicharz et al. 2025). Additionally, at 16 DAI, the activity of the synthetic cytokinin-responsive promoter TCS (two-component signaling sensor), which was strong in hypocotyl cells destined to become phloem tissues, was nearly undetectable in the infected plants (Singh et al. 2022). Collectively, these findings indicate that CK activity declines sometime after pathogen infection, potentially beginning with the onset of gall formation.

Plants perceive CKs through CHASE domain-containing histidine kinase receptors, which include three members in Arabidopsis, ARABIDOPSIS HISTIDINE KINASE2 (AHK2), AHK3, and AHK4 (Fig. 3). The binding of CKs results in autophosphorylation of the receptor, triggering a phosphorylation cascade that ultimately phosphorylates type-A and type-B response regulators (RRs). Phosphorylation of type-B RRs activates CK-responsive genes, including *CYTOKININ RESPONSE FACTORS* (*CRFs*), as well as type-A *RRs* that function as negative regulators of CK response (Keshishian and Rashotte 2015; Li et al. 2021). Multiple CRFs were differentially expressed in the hypocotyl phloem tissues of infected *B. napus* plants during early disease development, indicating potential modulation of CK activity (Blicharz et al. 2025). However, wild-type Arabidopsis at 16 and 26 DAI showed a general down-regulation of type-A RRs, supporting the idea of reduced CK later in disease development (Malinowski et al. 2016).

In *ipt1;3;5;7* quadruple mutants, which exhibit root growth defects due to severely reduced CK levels, the disease index was reduced, and the pathogen life cycle was slowed. However, these mutants still produced root galls and showed increased expression of type-A *RRs*, indicating potential activation of CK biosynthesis by the pathogen (Malinowski et al. 2016). Interestingly, *P. brassicae* contains two *IPT* homologs (Rolfe et al. 2016; Schwelm et al. 2015) and is likely capable of producing tZ (Müller and Hilgenberg 1986). Nevertheless, the potential contributions of pathogen-stimulated CK biosynthesis in clubroot development appear to be limited, as the growth defects caused by CK deficiency in the above quadruple mutants, such as the lack of vascular cambium development and secondary thickening, were not rescued (Malinowski et al. 2016).

Even though multiple studies suggest that CK activity increases before gall formation and decreases afterward, inconsistencies exist. In one Chinese cabbage study, zeatin (not differentiated as trans-zeatin and cis-zeatin) and CK-ribotide levels were generally lower in infected roots at 6, 13, and 21 DAI, while zeatin riboside levels were higher (Devos et al. 2005). In Arabidopsis, microarray analysis showed more CK biosynthesis and signaling-related genes downregulated than upregulated at 10 DAI, suggesting a potential down-regulation of CK action even at this early stage. However, further down-regulation was observed for most genes at 23 DAI (Siemens et al. 2006). Two of the three differentially regulated cytokinin oxidases, *CKX1* and *CKX6*, were also among the downregulated genes (Siemens et al. 2006). Cell-specific expression analysis using laser-capture microdissection also showed reduced expression of *CKX1*, *CKX3*, and *CKX7* in the central cylinder of infected roots at 14 DAI (Schuller et al. 2014). Since CKX enzymes mediate CK inactivation, lower *CKX* expression may result from reduced CK biosynthesis, or it could be a mechanism to increase active CK levels. Supporting the latter, overexpression of *CKX1* and *CKX3* reduced gall development, indicating that higher CK activity facilitates gall formation (Siemens et al. 2006).

While the expression of *CKXs* and many other CK-related genes is often affected by *P. brassicae* infection, the patterns vary across genes and developmental stages. In cabbage (*Brassica oleracea* var. *capitata* L.), where gall formation was not observed until 28 DAI, some *CKX* genes were upregulated, while others were downregulated in both susceptible and resistant cultivars at 7 and 28 DAI (Zhu et al. 2022). In Chinese cabbage, five *CKX* genes and multiple other CK-related genes (including histidine kinases) showed no clear expression patterns during early clubroot development. However, the expression of C*KX2*, *3,* and *6* generally increased at early stages and decreased by 35 DAI as galls enlarged (Laila et al. 2020). Microarray analysis in Arabidopsis also found differential expression of *CKXs*, *ARRs*, and several other CK-related genes based on tissue type within the root and plasmodial developmental stages in selected cells (Schuller et al. 2014).

Increased activity of existing meristems appears to drive root gall formation in *P. brassicae*-infected plants (Malinowski et al. 2012). While *CYCD3;1*, a key gene controlling G1/S cell cycle progression, is crucial for CK-induced cell proliferation (Shimotohno et al. 2021), it is not significantly affected by *P. brassicae* infection. Instead, other regulatory genes suggest cells remain longer in the proliferation stage, delaying cell cycle exit (Olszak et al. 2019). Currently, our understanding of CK-dependent cell cycle regulation is largely limited to CYCD3-dependent aspects (Shimotohno et al. 2021), leaving the importance of CKs in the observed shifts in cell cycle activity unclear. On the other hand, the *abcg14* mutant, which accumulates high levels of tZ and cZ in underground organs due to disrupted CK transport, showed no difference in disease progression and formed smaller root galls, indicating that elevated CK levels do not necessarily promote gall formation (Blicharz et al. 2025).

Given the role of CKs in suppressing protoxylem formation, the increased CK levels early in infection are suggested to play a role in the reduced xylem development seen in the infected plants (Malinowski et al. 2012; Osugi and Sakakibara 2015). Impaired water uptake caused by root deformities, antagonistic interactions between CK and the drought-induced hormone ABA, and the suppression of CK content and signaling during drought stress may contribute to the lower CK levels observed later in disease development (Cortleven et al. 2019; Huang et al. 2018).

Additionally, certain forms of CK can act as long-distance signals, transported from roots to shoots, to regulate growth in response to environmental factors like soil nitrogen availability (Abualia et al. 2022; Osugi et al. 2017). Developing root galls act as strong nutrient sinks and impair plant water uptake, resulting in stunted aboveground growth. A recent study in *B. napus* showed that *P. brassicae* infection alters the expression of genes involved in carbon and nitrogen partitioning in phloem cells, including those encoding nitrate, amino acid, and sugar transporters. Phloem sap collected from leaf petioles of infected plants showed a decrease in amino acid and sucrose content, along with elevated iP-type CK levels (Blicharz et al. 2025). Given these changes in CK levels, together with the role of CK in root-to-shoot signaling, it is possible that plants also modulate CK levels to coordinate root and shoot growth under conditions of skewed nutrient distribution. Therefore, while CK levels and activity appear spatially and temporally modulated to facilitate root gall formation, further studies focusing on these diverse aspects are needed to understand the exact roles of CKs in plant responses to *P. brassicae* infection.

## Salicylic Acid

The phenolic compound SA is a pivotal phytohormone in plant immunity, particularly against pathogens with biotrophic or hemibiotrophic lifestyles (Glazebrook 2005). SA is required for pathogen-associated molecular pattern (PAMP)-triggered immunity (PTI) and effector-triggered immunity (ETI), both of which activate a broad range of defense responses against pathogens, including the generation of reactive oxygen species, activation of defense-associated genes, and accumulation of antimicrobial compounds (Dempsey and Klessig 2017). SA is also important for stimulating immune responses in uninfected tissues, a process known as systemic acquired resistance (SAR), although it is not the mobile signal that travels from infected to uninfected tissues (Zhang and Li 2019). Beyond its role in immunity, SA plays crucial roles in regulating various growth and developmental processes and engages in crosstalk with other phytohormones (Peng et al. 2021).

Elevated SA levels have been reported in both resistant and susceptible host interactions with *P. brassicae*, although the increase is usually more pronounced in resistant responses. A study in *B. napus* found no significant changes in SA at earlier time-points, but an increase in the partially resistant cultivar starting at 35 DAI and the susceptible cultivar at 49 DAI (Prerostova et al. 2018). Another study involving different *B. napus* cultivars showed higher SA levels during both susceptible and resistant interactions at 21 and 28 DAI, with a substantially greater increase in the resistant interactions (Jayasinghege et al. 2023). In Arabidopsis, SA levels remained unchanged in the roots of the susceptible ecotype but significantly increased in the partially resistant ecotype at 14 DAI (Lemarié et al. 2015a). In susceptible Chinese cabbage, SA levels rose after inoculation and increased further as symptoms developed (Ji et al. 2021).

**Fig. 4:**
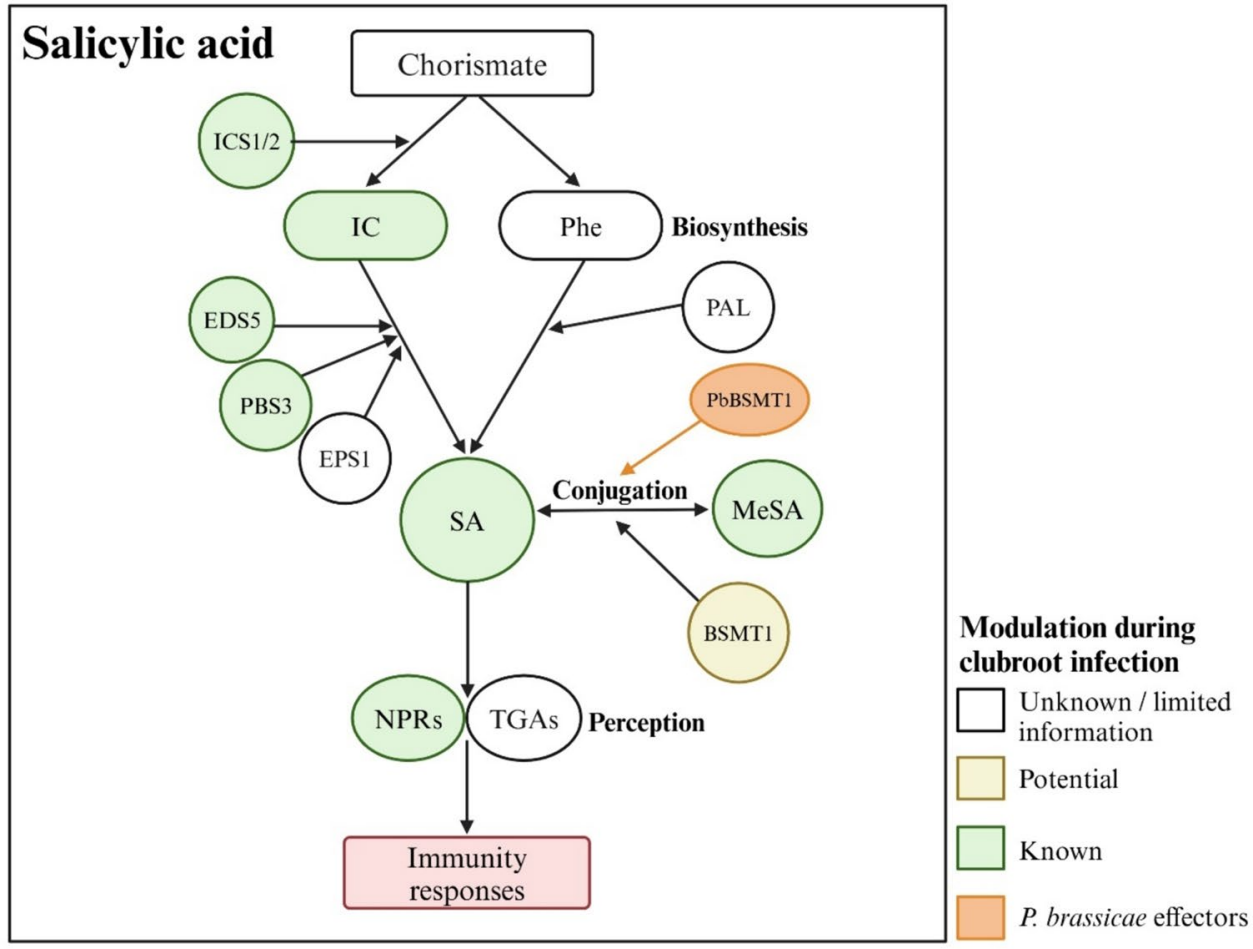
Salicylic acid (SA) and clubroot defense. SA is synthesized from chorismate via isochorismate (IC) or phenylalanine (Phe) by the isochorismate synthase (ICS) and phenylalanine ammonia-lyase (PAL) pathways, respectively. The ICS pathway likely predominates in Brassicaceae plants infected with *P. brassicae*. Key ICS pathway genes, *EDS5*, *PBS3*, and *EPS1*, are typically upregulated during *P. brassicae* infection, with resistant interactions typically showing stronger expression and higher SA levels. SA levels are further regulated by conversion into various forms, such as methyl SA (MeSA), catalyzed by BSMT1, with *P. brassicae* also harboring a functional BSMT homolog. In SA signaling, NPR receptors interact with TGA transcription factors to regulate SA-responsive gene expression. *P. brassicae* infection often enhances *NPR* expression, suggesting the activation of SA-mediated defense mechanisms

Plants synthesize SA from chorismate via the isochorismate synthase (ICS) and phenylalanine ammonia-lyase (PAL) pathways (Fig. 4). In Arabidopsis, the ICS pathway predominates (Peng et al. 2021) including during clubroot disease development (Lovelock et al. 2016). RNAseq studies indicate an overall activation of *ICS*-related biosynthesis genes compared to *PAL*-related genes during both the primary and secondary stages of *P. brassicae* infection (Galindo-González et al. 2020; Jia et al. 2017; Jubault et al. 2013; Zhou et al. 2020). Both susceptible and resistant interactions show increased *ICS1* transcript levels upon infection, though this increase may not be consistently detectable at all time-points (Ji et al. 2021; Jia et al. 2017; Jubault et al. 2013; Lovelock et al. 2016; Prerostova et al. 2018). The *ICS* homolog, *ICS2*, which plays a minor role in SA biosynthesis in Arabidopsis and is typically not induced by pathogen infection (Peng et al. 2021), also showed differential expression in *B. napus* during the secondary phases of infection (Galindo-González et al. 2020; Zhou et al. 2020).

The ICS pathway begins in the chloroplasts, where chorismate is converted to isochorismate (IC). The ENHANCED DISEASE SUSCEPTIBILITY5 (EDS5) protein then transports IC into the cytosol, where AvrPphB SUSCEPTIBLE 3 (PBS3) conjugates IC to glutamate (Glu) to form IC-9-Glu (Lefevere et al. 2020). This compound can then be converted to SA by ENHANCED PSEUDOMONAS SUSCEPTIBILITY 1 (EPS1) (Torrens-Spence et al. 2019) or spontaneously decompose into SA (Rekhter et al. 2019). Similar to *ICS1*, transcript levels of *EDS5* and *PBS3* are typically induced by pathogen infection (Peng et al. 2021). Increased expression of *EDS5* in response to *P. brassicae* has been reported in resistant *B. napus* (Galindo-González et al. 2020) and *B. rapa* (Jia et al. 2017). However, the Arabidopsis SA-deficient mutant *eds5-1* showed slightly reduced susceptibility, likely due to increased JA activity in this mutant (Lemarié et al. 2015a). Increased transcript levels of *PBS3* have also been reported during both susceptible and resistant interactions in *B. napus*, although resistant interactions maintained elevated levels longer (Galindo-González et al. 2020; Zhou et al. 2020). In *B. rapa, PBS3* transcript levels were significantly upregulated in the resistant but downregulated in the susceptible hosts at 30 DAI (Jia et al. 2017). RNAseq analysis in Arabidopsis showed initial activation of *PBS3* by 1 DAI (Zhao et al. 2017), indicating early activation of SA-mediated defense.

As free SA accumulates in the cytoplasm, its level is further modulated through chemical modifications, such as glycosylation, methylation, amino acid conjugation, and hydroxylation (Ding and Ding 2020). Glycosylation of SA produces inactive storage forms, such as salicylate glucose ester and SA 2-O-β-D-glucoside (SAG), which can be readily converted back to SA. Methylation produces methyl SA (MeSA), a volatile compound released by plants, while hydroxylation produces dihydroxybenzoic acids (DHBAs) (Ding and Ding 2020). Interestingly, some pathogens modulate SA metabolism to overcome plant defense mechanisms by reducing bioactive SA levels (Peng et al. 2021). Hormone analysis of resistant and susceptible responses to clubroot in *B. napus* found that the ratio of free SA to total conjugated SA was higher in the former (Jayasinghege et al. 2023). While Arabidopsis contains much lower levels of MeSA compared to SAG and DHABs (Peng et al. 2021), MeSA has gained more attention in clubroot disease research due to its fluctuating levels and the potential role of the pathogen in modulating its production.

The methylation of SA to MeSA is catalyzed by the methyltransferase BENZOIC ACID/SA CARBOXYL METHYLTRANSFERASE 1 (BSMT1), a member of the SABATH family of methyltransferases (Chen et al. 2003; Ding and Ding 2020). In *B. oleracea* and *B. napus*, *BSMT1* transcript levels were unchanged or downregulated in response to *P. brassicae* infection (Manoharan et al. 2016; Zhou et al. 2020). However, when incubated with deuterium-labeled SA, the roots of inoculated plants produced more MeSA than the non-inoculated controls (Ludwig-Müller et al. 2015). This increased methylation is postulated to be caused by *PbBSMT*, a *P. brassicae* gene with low homology to the plant SABATH family genes (Ludwig-Müller et al. 2015)*.* PbBSMT appears to act as a potent methyltransferase, capable of reducing SA levels more effectively than AtBSMT1 when overexpressed transgenically in Arabidopsis (Djavaheri et al. 2019). Overexpression of *PbBSMT* also rendered Arabidopsis plants more susceptible to clubroot (Bulman et al. 2019; Djavaheri et al. 2019), indicating that PbBSMT can function as a pathogen effector to overcome SA-mediated host defense.

The binding of SA to NONEXPRESSOR OF PATHOGENESIS-RELATED GENES (NPR) proteins initiates downstream plant responses (Zhang and Li 2019) (Fig. 4). NPR proteins interact with TGACG motif-binding (TGA) transcription factors to regulate SA-responsive gene expression. NPR1 acts as a transcriptional activator in the presence of SA, activating defense-related genes, while NPR3 and NPR4 function as repressors when SA levels are low (Peng et al. 2021). Pathogen infection and SA treatment often increase the expression of *NPRs* (Peng et al. 2021), and increased transcript or protein levels have also been reported in responses to *P. brassicae* (Adhikary et al. 2022; Galindo-González et al. 2020; Ji et al. 2021; Jubault et al. 2013; Ning et al. 2019; Zhou et al. 2020). However, clubroot severity in Arabidopsis *npr1-2* was similar to the wild-type (Lemarié et al. 2015a), while disease severity increased in *npr1-1* (Chen et al. 2016). Another study with an unspecified *npr1* mutant reported reduced gall severity but also reduced shoot weight (Lovelock et al. 2016). While the *npr1* mutants used in these studies are expected to be SA signaling-deficient, it remains unclear if the observed differences in disease susceptibility are mutation-dependent or due to the complex regulation of SA action.

SA treatments, whether by dipping seedling roots in an SA solution or via repeated soil or foliar applications, significantly reduced clubroot severity in Arabidopsis (Agarwal et al. 2011; Chen et al. 2016; Lemarié et al. 2015a). Foliar application of SA in *B. napus* (Prerostova et al. 2018), direct application to *P. brassicae*-infested soil in Pak choi (*B. rapa* ssp. *chinensis* Makino) (Xi et al. 2021), and a root drench application of SA in broccoli (*B. oleracea* var. *italica*) (Lovelock et al. 2013) also reduced clubroot severity. Despite the commonly described antagonism between SA and JA in plant defense, combined JA and SA treatments also reduced disease severity in broccoli (Lovelock et al. 2013). Furthermore, SA applied to *B. napus* was readily metabolized after application, indicating effective SA utilization (Prerostova et al. 2018). This rapid uptake and metabolism of SA could also explain why some SA applications, particularly those involving a single dose, did not reduce clubroot severity (Ji et al. 2021; Lovelock et al. 2016).

Collectively, SA accumulation observed predominantly in resistant interactions and the reduced clubroot severity in plants treated with SA underscores its significance as a regulator of plant defense. Although the expression patterns of genes involved in SA biosynthesis and regulation are not always consistent, they indicate active modulation of SA in infected plants. Evidence suggesting *P. brassicae* manipulation of plant-host SA levels further emphasizes its need to overcome SA-mediated plant defense. Unfortunately, the increased SA levels in defense are often associated with trade-offs in growth and development (van Butselaar and Van den Ackerveken 2020), as also found in some of the above studies, including delayed and reduced flowering (Agarwal et al. 2011) and reduced shoot and root weights (Lovelock et al. 2013). As a result, direct modulation of SA to enhance defense has practical limitations, but a deeper understanding of the underlying mechanisms of SA-mediated defense could open new avenues for improving clubroot resistance.

## Jasmonates

JAs are a group of lipid-derived signaling molecules that include jasmonic acid (JA), its biologically active form JA-Ile, and other JA derivatives (Wasternack and Song 2016). JAs regulate various plant growth and development processes, and abiotic stress responses, and play a crucial role in plant defense against pathogens and herbivore attacks (Huang et al. 2017). While JAs are primarily associated with host plant responses to necrotrophic pathogens, evidence suggests that their influence may also extend to some biotrophic pathogens (Antico et al. 2012), including *P. brassicae* (Lemarié et al. 2015a).

Multiple studies have reported alterations in root JA levels during clubroot development. In *B. napus*, one study found reduced JA and JA-Ile levels during rapid root gall enlargement. Resistant interactions, on the other hand, showed an increase in JA and JA-Ile levels during later stages of rapid gall enlargement (28 DAI) (Jayasinghege et al. 2023). A second study initially observed a slight reduction followed by an increase in JA levels after *P. brassicae* infection (Xu et al. 2018). A third study involving a susceptible and a partially resistant *B. napus* cultivar reported increased JA levels at various time-points, ranging from 2 to 49 DAI, irrespective of clubroot susceptibility (Prerostova et al. 2018). Increased levels of JA or JA-Ile have also been reported in clubroot-susceptible or -resistant cultivars or lines of Arabidopsis (Gravot et al. 2012; Lemarié et al. 2015a), *B. rapa* (Grsic et al. 1999; Wei et al. 2021), and *Matthiola incana* (Xu et al. 2018). However, some of these studies also found reduced JA levels at specific time-points (Grsic et al. 1999; Wei et al. 2021).

The above studies suggest that infected plants do not consistently accumulate JAs in response to *P. brassicae* infection, but JA modulation occurs in both resistant and susceptible host responses. A key question remains whether these changes, particularly increased JA levels, contribute to clubroot defense. Interestingly, a single JA foliar treatment to *B. napus* increased clubroot disease severity (Prerostova et al. 2018). In contrast, repeated applications of methyl jasmonate (MeJA) to the plant crown, which is converted to JA-Ile within plant tissues (Wasternack and Song 2016), reduced pathogen levels in both Arabidopsis Bur-0 and Col-0 ecotypes and alleviated clubroot symptoms in Col-0 (Lemarié et al. 2015a).

More compelling evidence supporting the role of JAs in clubroot defense comes from studies on the GH3 family member *JASMONATE RESISTANT 1* (*JAR1; GH3.11*), a JA-amino synthetase that converts JA to its biologically active form, JA-Ile. Loss-of-function *jar1-1* and *jar1-8* mutants in Arabidopsis result in significantly lower JA-Ile levels (Staswick and Tiryaki 2004), and *jar1* mutants consistently show more severe clubroot development (Gravot et al. 2012; Lemarié et al. 2015a; Siemens et al. 2002). One study, however, found no difference (Agarwal et al. 2011), possibly due to the already high susceptibility (100%) of the wild-type plants. *P. brassicae* infection reduces *JAR1* expression in Arabidopsis (Jahn et al. 2013; Jubault et al. 2013; Schuller et al. 2014; Siemens et al. 2006), canola (*B. napus* var. *napus* ‘Brutor’), and a partially clubroot-resistant rutabaga cultivar (*B. napus* ssp. *napobrassica* ‘Laurentian’) (Galindo-González et al. 2020). Moreover, *JAR1* expression was lower in galled roots of kohlrabi (*B. oleracea* var. *gongylodes*) than in symptomless roots (Ciaghi et al. 2019). Reduced *JAR1* expression in the roots of infected plants, lower JA-Ile levels observed in some susceptible infected plants, and the increased susceptibility of *jar1* mutants suggest that reduced JA activity due to lower JA-Ile levels contributes to clubroot development. Fluctuations in JA levels observed in various studies may reflect the plant’s efforts to synthesize more JA-Ile as a defense response.

**Fig. 5:**
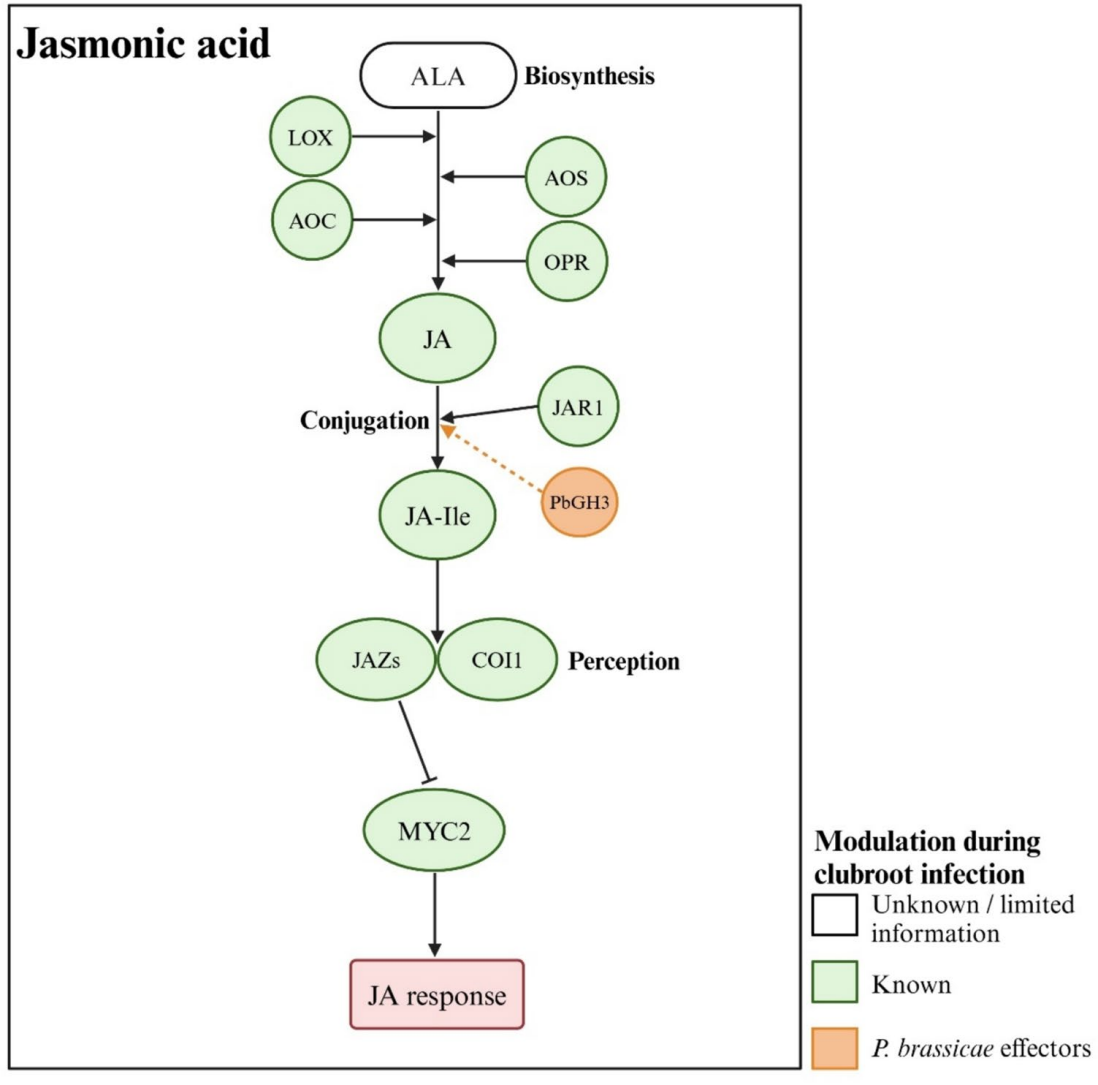
Jasmonic acid (JA) dynamics in clubroot defense. JA is synthesized from α-linolenic acid (ALA) through the activity of key enzymes, LOX, AOS, AOC, and OPR. The GH3 family member JAR1 converts JA into its active form, JA-Ile. Although *P. brassicae* also possesses a GH3 homolog, it is unlikely to play a major role in this process. JA perception by COI1 and JAZ in the presence of JA-Ile leads to the degradation of JAZ, allowing MYC2 and other bHLH family transcription factors to regulate JA-responsive gene expression. *P. brassicae* infection modulates JA levels and the expression of biosynthesis- and signaling-related genes, while *jar1* mutants show increased disease susceptibility, supporting a defensive role for JA against the clubroot pathogen. Pointed and flat-tipped arrows indicate stimulation and inhibition, respectively. Solid lines represent well-characterized processes, while dashed lines denote processes that are not yet well understood

Plants synthesize JA from the omega-3 fatty acid α-linolenic acid (ALA) via a pathway involving multiple enzymes, including lipoxygenases (LOX), allene oxide synthases (AOS), allene oxide cyclases (AOC), and 12-oxo-phytodienoic acid reductases (OPR) (Ghorbel et al. 2021; Wasternack and Song 2016) (Fig. 5). In a clubroot-resistant rutabaga (*B. napus* var. *napobrassica*) cultivar, *P. brassicae* infection downregulated an *AOS* homolog, three *AOC2s*, and two *OPR1s* at 7 DAI, while they were upregulated in a susceptible cultivar. However, later time-points indicated a general down-regulation of JA biosynthesis genes in both cultivars (Zhou et al. 2020). In an Arabidopsis study involving two *P. brassicae* pathotypes, *LOX2* expression decreased in partially resistant interactions at early disease development stages, while *LOX2* and *AOS* expression increased in susceptible interactions (Jubault et al. 2013). In kohlrabi, symptomless inoculated roots exhibited down-regulation of *AOC* and both up-regulation and down-regulation of *LOX* genes compared to roots with galls (Ciaghi et al. 2019). These findings are consistent with hormone profiling data indicating temporal and spatial modulations of JA levels during clubroot disease development.

JA signaling functions similarly to auxin, where the CORONATINE INSENSITIVE1 (COI1) and JASMONATE-ZIM DOMAIN (JAZ) proteins function as coreceptors. JAZ proteins repress MYC2 and other basic helix–loop–helix (bHLH) family of transcription factors that regulate JA-responsive gene expression (Fig. 5). JA-Ile facilitates the interaction between JAZ and COI1, leading to the ubiquitination and degradation of JAZ repressors, allowing MYC2 and other transcription factors to regulate JA-responsive genes (Howe et al. 2018). A transcriptomic analysis in *B*. *rapa*, comparing susceptible and resistant reactions to two *P. brassicae* pathotypes, revealed higher expression of four *COI1* homologs and a *MYC2* homolog, along with reduced expression of 11 *JAZ* repressors in the resistant compared to the susceptible responses at 8 DAI. These expression patterns, also confirmed by qPCR analysis for most of the genes, indicate higher JA activity in resistant reactions at early stages after pathogen inoculation (Fu et al. 2019). Furthermore, microarray and qPCR analysis in the central cylinder of Arabidopsis roots showed reduced expression of *COI1* and increased expression of three *JAZ* repressors, indicating reduced JA activity in response to susceptible interactions (Schuller et al. 2014).

Overall, JAs appear to play a role in defense against *P. brassicae*, with lower JA activity likely contributing to more severe disease development. The fluctuations in JA and JA-Ile profiles, along with varying expression patterns of JA biosynthesis and action genes, as well as other discrepancies observed in various studies, could be attributed to multiple factors. As the costs associated with defense mechanisms negatively impact plant growth and development, JA biosynthesis and action are restricted to specific periods (He et al. 2022; Howe et al. 2018). Therefore, while JA likely contributes to clubroot defense, the fluctuations observed may reflect the plant’s efforts to mitigate the adverse effects of constitutively high JA levels. Plants also induce JA biosynthesis in response to wounding (Huang et al. 2017). It has been suggested that some of the changes in JA levels observed after *P. brassicae* infection may result from a wound response triggered by the digestion of host cell walls during cortical invasion (Blicharz et al. 2025). Consistent with this idea, transcriptomic analyses in *Brassica* species show up-regulation of genes associated with wounding and reactive-oxygen-species production during early disease development (Blicharz et al. 2025; Ce et al. 2024).

JA is also known to interact with SA signaling, with these interactions generally being antagonistic (Hou and Tsuda 2022). Many biotrophic and hemibiotrophic pathogens exploit this antagonism by deploying effectors to activate the JA response, thereby suppressing SA-mediated defense (Howe et al. 2018), though no evidence to date suggests such a strategy by *P. brassicae*. Nonetheless, SA can also inhibit JA activity through various means, including modulation of transcription factor availability and activity. A prominent example is the SA-inducible WRKY transcription factors, many of which negatively regulate JA-responsive genes (Caarls et al. 2015). Therefore, altered SA levels in *P. brassicae*-infected plants may also influence the JA pathway and the plant response to JA treatments.

## Ethylene

The gaseous plant hormone ethylene, in addition to its various regulatory roles in plant growth and development, plays a crucial role in plant responses to both biotic and abiotic stresses (Dubois et al. 2018). In the context of biotic stress, ethylene is widely recognized for its synergy with JA in defense against necrotrophs. In some instances, ethylene can also positively or negatively influence SA-mediated defense (Broekgaarden et al. 2015). Several studies on clubroot disease have also explored whether ethylene plays a role in plant defense against *P. brassicae*.

**Fig. 6:**
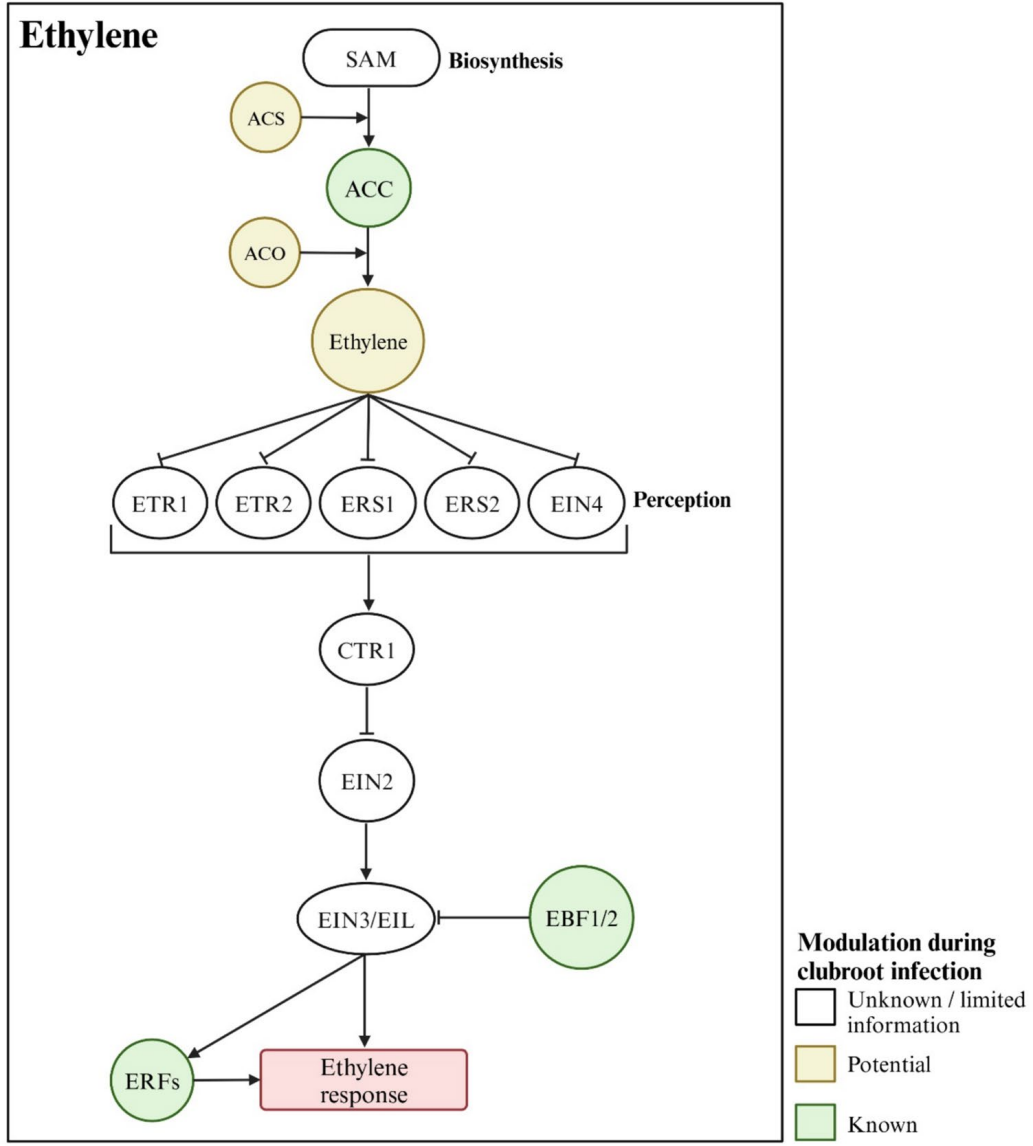
Ethylene-mediated modulation of clubroot disease. Ethylene is synthesized from S-adenosylmethionine (SAM), which is converted to 1-aminocyclopropane-1-carboxylic acid (ACC) by ACS and then to ethylene by ACO. Changes in ACC levels in response to *P. brassicae* infection suggest alterations in ethylene biosynthesis. Ethylene is perceived by the receptors ETR1, ETR2, ERS1, ERS2, and EIN4, which inactivate CTR1, triggering downstream signaling through EIN2. EIN2 inhibits EBF1 and EBF2, stabilizing the EIN3/EIL transcription factors. These transcription factors regulate ethylene-responsive genes, including *ERFs*, which control downstream ethylene responses. Mutants of key ethylene signaling genes show no difference in disease susceptibility at high inoculum densities but show increased susceptibility at low inoculum densities, suggesting a positive role for ethylene in clubroot defense. Pointed and flat-tipped arrows indicate stimulation and inhibition, respectively

Plants synthesize ethylene via a two-step pathway. In the first step, S-adenosylmethionine (SAM) is converted to 1-aminocyclopropane-1-carboxylic acid (ACC) by ACC synthase (ACS). Subsequently, ACC is oxidized to ethylene by ACC oxidase (ACO) (Pattyn et al. 2021; Fig. 6). While ACO activity may regulate the rate of ethylene biosynthesis under specific conditions, the level of ACC is generally regarded as the rate-limiting step and is frequently used to estimate alterations in ethylene biosynthesis (Ishikawa et al. 2011; Jayasinghege et al. 2017; Pattyn et al. 2021). In *B. napus*, *P. brassicae* infection had no clear effect on the root ACC level in either susceptible or resistant cultivars during early disease development, but its levels increased later in both cultivars. While inoculated susceptible plants had the highest ACC content, the increase was more pronounced in resistant plants compared to their respective non-inoculated controls (Jayasinghege et al. 2023). Similarly, in turnip (*B. rapa* ssp. *rapa*), ACC levels and ACO activity (normalized to tissue weights) were not affected by *P. brassicae* infection at early time-points but increased at later stages (Ishikawa et al. 2011). In contrast, inoculation of Chinese cabbage did not impact ACC levels when evaluated at 6, 13, and 21 DAI (Devos et al. 2005), whereas Arabidopsis showed decreased ACC levels at 10 and 20 DAI, with no changes at later time-points (Knaust and Ludwig-Müller 2013).

The above observations can be interpreted in different ways. High ACC levels, along with high ACO activity, indicate increased ethylene biosynthesis. While elevated ACC levels can be indicative of higher ethylene production, lower ACC levels due to increased ACO activity may also reflect high ethylene production. Supporting this latter possibility, consistently upregulated expression of an *ACO* gene was reported in Arabidopsis upon *P. brassicae* inoculation (Knaust and Ludwig-Müller 2013). Similarly, another study reported increased expression of one *ACS* and one *ACO* gene during a partially resistant interaction (Jubault et al. 2013). However, more detailed evaluations are required to determine the impact of these expression changes on ethylene biosynthesis, as both *ACS* and *ACO* belong to multigene families (Pattyn et al. 2021). Determining ethylene levels, along with the expression patterns of ethylene biosynthesis genes and ACC levels, would help clarify the effect of *P. brassicae* infection on ethylene biosynthesis in infected roots.

The ethylene signaling pathway in Arabidopsis includes five receptors: ETR1 (ETHYLENE RESPONSE 1), ETR2, ERS1 (ETHYLENE RESPONSE SENSOR 1), ERS2, and EIN4 (ETHYLENE INSENSITIVE 4). In the absence of ethylene, these receptors activate the repressor protein CONSTITUTIVE TRIPLE-RESPONSE 1 (CTR1), which inhibits ethylene signaling (Binder 2020). When ethylene binds to the receptors, they and CTR1 are deactivated, initiating downstream signal transmission through EIN2. This leads to the degradation of mRNAs coding for the negative regulators EIN3 BINDING F-BOX 1 (EBF1) and EBF2, stabilizing the EIN3 and EIN3-like (EILs) transcription factors. The stabilized EIN3/EILs regulate the expression of ethylene-responsive genes, including ETHYLENE RESPONSE FACTORS (ERFs) that control additional ethylene-responsive genes (Binder 2020); Fig. 6).

Despite being important components of the ethylene signaling pathway, the *etr1* and *ein4* receptor mutants (Siemens et al. 2002), as well as *ein2*, *ein3*, and *eil1* mutants (Alix et al. 2007; Siemens et al. 2002), showed no significant changes in clubroot susceptibility compared to the wild-type at high inoculum densities (10^6^–10^7^ resting spores/ml). However, *ein3eil1* double mutants (Wang et al. 2022b), and *ein2*, *ein3-1*, *ein4,* and *etr1-1* mutants at lower inoculum densities (Knaust and Ludwig-Müller 2013), exhibited increased clubroot susceptibility. These observations indicate that reduced ethylene activity enhances clubroot development. Further supporting a role of ethylene in disease suppression, the ethylene overproducing *ACS5* mutant *eto2* (*ethylene overproducer2*) showed slightly reduced clubroot susceptibility in one study (Knaust and Ludwig-Müller 2013), though an earlier study reported no difference in susceptibility (Alix et al. 2007).

*P. brassicae* infection also influences the plant-host ethylene-related gene expression. Transcriptomic analysis in rutabaga (*B. napus* ssp. *rapifera*) revealed increased expression of *ERF* genes in resistant but not susceptible interactions (Zhou et al. 2020). Similarly, in Chinese cabbage, multiple ethylene receptor homologs, *EBF1* and *EBF2* homologs, and *ERF* genes were upregulated in resistant interactions but not susceptible ones (Fu et al. 2019). Since the expression of all these genes is induced by ethylene (Jayasinghege et al. 2017; Müller and Munné-Bosch 2015), these changes provide further evidence that ethylene activity is likely high in plants showing resistance. Taken together with increased susceptibility of ethylene signaling mutants and shifts in ethylene biosynthesis upon *P. brassicae* infection, this evidence supports a role for ethylene in clubroot defense.

Ethylene is also an important player in plant hypoxia responses, as it regulates the expression and enhances the stability of group VII ERFs (ERFVII). Under hypoxic conditions, ERFVII transcription factors activate a core set of genes that enable plants to adapt to low-oxygen environments. Among these genes, *PYRUVATE DECARBOXYLASE* (*PDC*) encodes a key enzyme necessary for ethanol fermentation, allowing plants to maintain glycolytic flux (Hartman et al. 2021). Clubroot disease development is influenced by soil water content, with poor aeration caused by excessive water resulting in reduced disease severity (Gravot et al. 2016a). However, even in well-aerated soil, the secondary infection phase induces hypoxia-response genes, including *PDC1* and *PDC2,* in Arabidopsis (Gravot et al. 2016b). Mutants lacking *PDC1* and *PDC2,* as well as those deficient in ERFVII transcription factor activity, showed reduced clubroot symptoms. In contrast, mutants with stabilized ERFVII transcription factors that show constitutive hypoxia responses exhibited more severe symptoms (Gravot et al. 2016b). Although no direct link has been observed between ethylene activity and hypoxia responses during clubroot development, the important role of ethylene in hypoxia responses, along with the influence of hypoxia responses on disease progression, suggests that it would be worthwhile to explore whether ethylene’s role in clubroot development is also mediated through this interplay.

## Abscisic Acid

ABA is a central regulator of plant responses to abiotic stress, particularly drought stress, which causes a rapid increase in ABA levels. ABA also regulates various growth and development processes and modulates plant defense responses to pathogens (Berens et al. 2017; Chen et al. 2020). While ABA helps protect against certain pathogens by closing the stomata to prevent their ingress, it suppresses defense against many others by inhibiting SA-mediated responses (Berens et al. 2017). Interestingly, some pathogens appear to exploit these antagonistic interactions to their advantage by producing ABA, inducing ABA biosynthesis in plants, or inhibiting ABA catabolism to overcome SA-mediated defenses (Berens et al. 2017; Cao et al. 2011).

Root galls in *P. brassicae*-infected plants impair water uptake, likely influencing ABA accumulation. In Chinese cabbage and canola, ABA levels remain unchanged during the first two weeks after inoculation, when the impact of root galls on water uptake is minimal. However, by the third week, ABA levels increase with the emergence of distinct root galls (Devos et al. 2005; Jayasinghege et al. 2023). Clubroot-resistant canola also shows a slight increase in ABA levels by 3 weeks post-inoculation, possibly due to the formation of small galls (Jayasinghege et al. 2023). Plants regulate ABA levels by fine-tuning the balance between biosynthesis and catabolism. ABA is predominantly catabolized first to phaseic acid (PA), then to dihydrophaseic acid (DPA) (Seo and Marion-Poll 2019). In susceptible canola plants, but not in resistant ones, ABA, PA, and DPA levels increased in the roots by the third week after inoculation, indicating increased ABA biosynthesis and catabolism (Jayasinghege et al. 2023).

The trends observed in ABA levels in plants infected with *P. brassicae* are not without exceptions. In canola, one study found no apparent changes in ABA levels in the roots or leaves at most of the time-points spanning early to late disease development (Prerostova et al. 2018). The *NINE-CIS-EPOXYCAROTENOID DIOXYGENASE 3* (*NCED3*) gene, one of the five *NCEDs* in Arabidopsis that regulate ABA biosynthesis and the primary NCED gene induced by dehydration (Seo and Marion-Poll 2019), showed no differential expression with *P. brassicae* infection; however, expression of the other four homologs was not reported (Prerostova et al. 2018).

Additionally, an increase in ABA biosynthesis could negatively influence SA-mediated defense mechanisms. Such impacts could further facilitate disease progression, a scenario observed in multiple other plant–pathogen interactions (Choudhary and Senthil-Kumar 2022; Xu et al. 2013; Yasuda et al. 2008). Therefore, it would be interesting to explore whether root galls benefit the pathogen not only by providing space for multiplication, but also by indirectly suppressing SA-mediated host defense.

## Brassinosteroids

BRs are a class of plant steroids that play roles in cell division and elongation, xylem differentiation, biotic and abiotic stress responses, and other regulatory functions (Nolan et al. 2020). Among the over 70 BR compounds in plants, brassinolide is the most common and active (Wei and Li 2020). The Arabidopsis BR-deficient mutant *de-etiolated 2-1* (*det2-1*) showed reduced clubroot disease severity (Siemens et al. 2002), while a high-throughput proteomics analysis in Chinese cabbage found elevated levels of BR biosynthesis-related proteins such as cycloartenol synthase (CAS1) and cytochrome P450 51G1 (CYP51G1) in infected plants (Su et al. 2018). Treatment with U18666A, a CAS1 activity inhibitor, decreased disease severity, though it also impaired overall plant growth, and its effect on BR levels is unclear (Su et al. 2018). Nonetheless, these observations suggest a positive role for BRs in root gall formation.

A microarray analysis of Arabidopsis cells with large secondary plasmodia showed that the expression of genes related to BR metabolism and signal transduction generally increased toward the later stages of root gall development (Schuller et al. 2014). While treatments with an active BR in the form of a brassinolide isomer did not influence clubroot development in these plants, treatment with the BR biosynthesis inhibitor propiconazole resulted in reduced disease severity, further supporting a role of BRs in root gall formation (Schuller et al. 2014).

Plants perceive BRs using plasma membrane-localized receptors, BR INSENSITIVE1 (BRI1), BRI1-LIKE1 (BRL1), and BRL3. Upon binding to receptors, BR acts as a “molecular glue” to recruit the coreceptor BRI1-ASSOCIATED KINASE1 (BAK1). This interaction initiates a signaling cascade that eventually activates BRI1-EMS-SUPPRESSOR1 (BES1) and BRASSINAZOLE-RESISTANT1 (BZR1) family transcription factors, which regulate BR-responsive gene expression (Nolan et al. 2020). When tested for clubroot susceptibility, the BR receptor mutant *bri1-6* showed reduced disease severity. Disease severity was not affected in the double-mutant *bri1-5 bak1-1*, but the percentage of plants in the higher disease class was lower, suggesting a similar trend (Schuller et al. 2014).

Overall, these data indicate a role for BR in root gall formation, potentially through the stimulation of cell division and elongation, key mechanisms of BR-mediated plant growth regulation (Nolan et al. 2020). BRs also engage in antagonistic interactions with ABA to modulate plant responses to drought stress (Nolan et al. 2020). The BES1 transcription factors inhibit many drought stress-induced genes, while their activity is reduced under drought stress conditions through mechanisms mediated by ABA (Jiang et al. 2019; Nolan et al. 2020). These antagonistic interactions, combined with increased ABA levels upon gall formation, may obscure any positive role BRs might play in disease development. Therefore, a detailed assessment of the roles of BRs both before and during gall formation is important for gaining a deeper understanding of their involvement in this process.

## Gibberellins

GAs are a group of plant hormones that play central roles in regulating vegetative and reproductive development. They are prominent players in stimulating cell expansion and, in some instances, promoting cell division (Hedden and Sponsel 2015). Despite these abilities, which make GAs potential regulators of root gall formation, a few studies have investigated their role in clubroot development. The application of the GA biosynthesis inhibitor chlormequat chloride did not affect root gall formation in Arabidopsis, but prohexadione calcium did reduce gall formation (Päsold and Ludwig-Müller 2013). While one microarray analysis in Arabidopsis indicated an increase in GA-related genes (Siemens et al. 2006), a later study found no significant increase at the same time-point (Schuller et al. 2014). Therefore, the existing literature, although limited, suggests that GAs do not play a significant role in modulating clubroot development.

## Summary

**Fig. 7:**
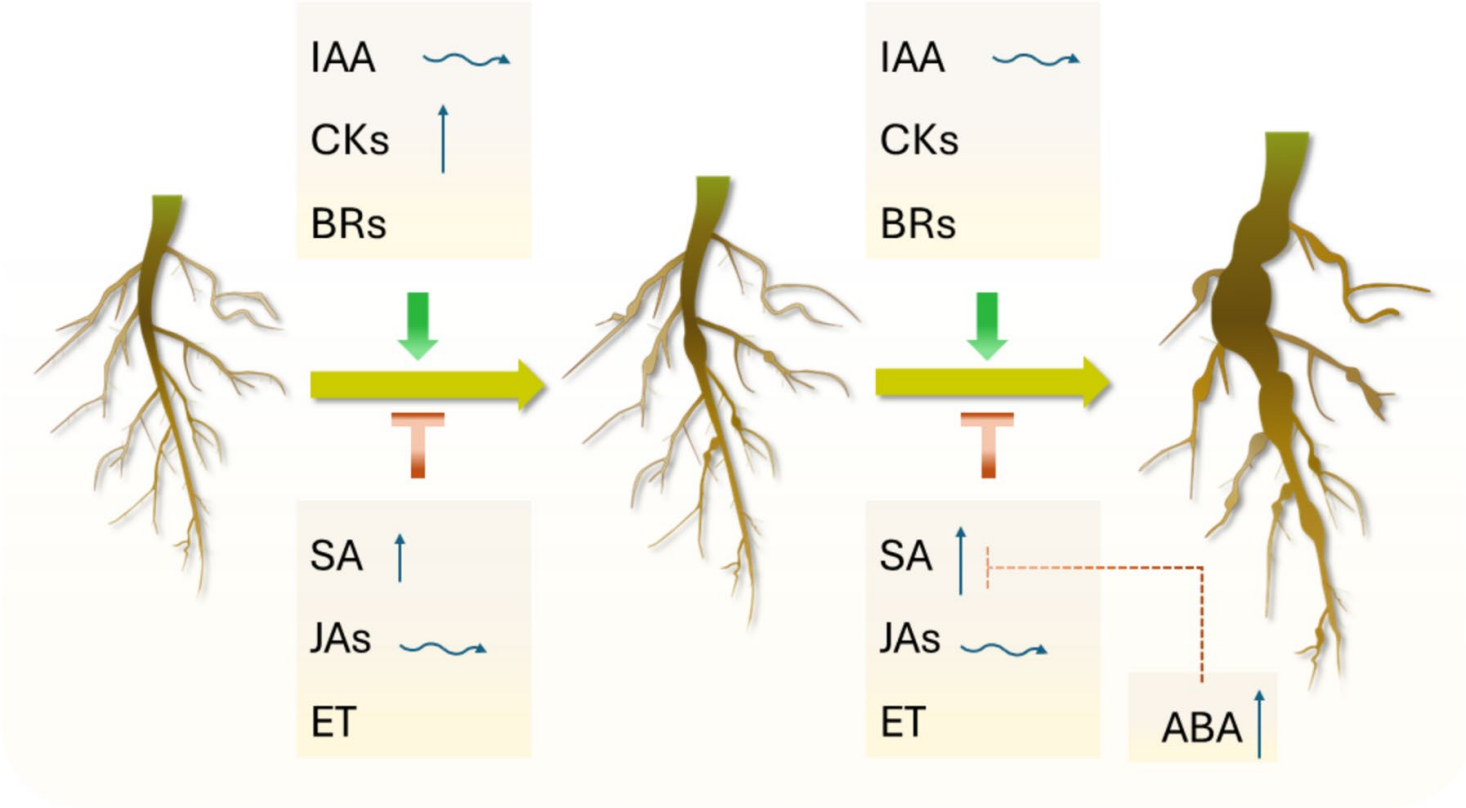
Hormonal regulation of clubroot disease development. Auxin (IAA), cytokinins (CKs), and brassinosteroids (BRs) are likely positive regulators of disease development (shown in green pointed arrows). Conversely, salicylic acid (SA), jasmonates (JAs), and ethylene (ET) contribute to plant defense or restrict gall formation (shown in red solid flat tip arrows). While trends are not always consistent, they generally indicate increased (blue arrows pointing up) CK levels, particularly at early stages, and fluctuating (blue-waved arrows) IAA levels in infected plants. SA levels also rise, with a more prominent increase at later stages and in resistant interactions, while JA levels appear variable. In later stages of infection, abscisic acid (ABA) production increases, which may help plants cope with reduced water uptake but may also favor the pathogen by interfering with SA-mediated defense (dashed flat tip arrow). Other hormone pathways appear to be mostly unaffected, or insufficient data are available to establish a general pattern

Most plant hormone pathways are affected by *P. brassicae* infection. These pathways enhance plant defense and associated stress responses, but in some cases, they are exploited by the pathogen to create conditions that are more favorable for disease progression. Changes in hormone levels, signaling, biosynthesis, and metabolism, alongside the responses of genetic mutants, provide evidence generally pointing to IAA, CKs, and BRs as potential facilitators of root gall development (Fig. 7). SA, on the other hand, appears to be the most prominent plant defense hormone against *P. brassicae*, while ethylene and JAs can also be categorized as defense hormones that likely contribute to restricting gall formation. Susceptible plants are likely to experience drought stress as root galls develop, leading to induced ABA biosynthesis. Given ABA’s role as a suppressor of SA-mediated defense, increased ABA levels may further assist pathogen growth and multiplication in the host.

Our understanding of the hormonal regulation of clubroot disease development and the associated stress responses has progressed significantly in the last few decades, but much remains to be elucidated. The influence of genetic and environmental factors, different *P. brassicae* pathotypes, stages of disease development, interactions among different hormones, and localized changes in hormonal action all contribute to the complexity of host–pathogen interactions. This complexity makes it difficult to generate a clear picture of the roles played by hormonal network responses during clubroot development. Future research targeting multiple host–pathogen combinations, developmental stages, the confirmation of gene expression changes observed in transcriptomic studies through more quantitative analysis, such as qPCR, and tissue-specific investigations will aid in resolving some of these complexities.
